# Measurement System for the Environmental Load Assessment of the Scale Ship Model

**DOI:** 10.3390/s23010306

**Published:** 2022-12-28

**Authors:** Anna Miller, Andrzej Rak

**Affiliations:** Faculty of Electrical Engineering, Gdynia Maritime University, 81-87 Morska Str., 81-225 Gdynia, Poland

**Keywords:** wind and wave parameters measurement, environmental disturbances, modeling of the ship motion process, ship scale model, microprocessor devices

## Abstract

The forces and moments acting on a marine vessel caused by the wind are most often modeled based on its speed measured at a standard 10 m above the sea level. There exist numerous well-known methods for modeling wind speed in such conditions. These models, by nature, are inadequate for simulating wind disturbances for free-running scale ship models sailing on lakes. Such scale models are being used increasingly for design and testing modern ship motion control systems. The paper describes the hardware and methodology used in measuring wind speed at low altitudes above the lake level. The system consists of two ultrasonic anemometers supplemented with wave sensor acting as a capacitor immersed partially in the water. Obtained measurement results show clear similarity to the values gathered during full-scale experiments. Analysis of the power spectral density functions of turbulence measured for different mean wind speeds over the lake, indicates that, at the present stage of research, the best model of wind turbulence at low altitude above the lake level can be obtained by assembling four of the known, standard turbulence models.

## 1. Introduction

Analyses of the marine wind and waves in a context of sea voyages have a long history. These phenomena have a fundamental influence on the marine vessels’ motions. Ancient sailors assessed wind strength and direction by just observing ribbons or cords tightened to the ships rigging. Waves were classified according to the physical phenomena that accompanied them.

In the 20th century, when the sailing ships were replaced by the vessels with mechanical propulsion, the role of the wind, seen from the seafarer’s perspective, changed from the driving force to the main source of disturbances. Intensive development of the automatic control systems applied to the ship steering in the second half of this century required deepened knowledge of the nature of environmental forces acting on the vessel. Numerous scientific projects were conducted in this area especially in the 1960s and 1970s. As a result of this effort, a few standard models of the wind and waves in a marine environment were proposed. This article covers the topic of wind disturbances. Modeling of the waves phenomena will be discussed in the separate publication.

The influence of wind on a seagoing vessel is most often modeled in a form of additive external forces and moments [1]. In the classical approach, they are determined as the functions of the average apparent wind velocity, which is determined as the difference between the ship’s own velocity and the velocity of the blowing wind [2,3] It is assumed that due to her large inertia, the ship will not react to the significant extent to the short-term changes of the wind speed and direction. This approach is natural for the synthesis of the control systems of the ship going in open waters, or in areas where a slight change in the course or position of the craft does not affect its safety. However, in the case of precise motion control at low speed, in the close presence of other objects (for example, during maneuvers in ports, locks or in time of ship-to-ship mooring operations), a short-term change in wind speed may lead to hazardous ship maneuvers [4,5]. To prevent such situations, different methods are used to compensate the impact of disturbances, for example by an additional feed-forward control signal dependent on the measured wind speed value. Hence, for such systems, simulation models of wind disturbances are supplemented by components modeling fast changes in their speed [6]:

(1) $$\tilde{V}\left(z,t\right)=\bar{U}\left(z\right)+v\left(z,t\right),$$

where $\tilde{V}\left(z,t\right)$ indicates instantaneous wind speed, $\bar{U}\left(z\right)$ indicates its mean value, v(z,t) represents the wind speed variations, *z* is measurement height and *t* denotes a time.

Short-term wind speed fluctuations, lasting from few seconds to one minute, are called wind gusts or turbulence. They are modeled as a random process, described by its spectral density function. This relation is determined empirically from measurements of wind speed in the environment for which the disturbance model is examined. Previous research in this area has resulted in the creation of a number of widely accepted models of turbulence. They are presented in Section 4.

Due to the physical phenomena in the boundary layer, the wind nature changes along the altitude above the sea level. Most frequently, the wind speed and direction models for the marine environment are prepared based on the data measured in one of the three zones:

- Around 100 m and more above a sea level, which is typical height for the offshore wind turbines analysis [7];
- In the span of 30–70 m above sea level appropriate for the off-shore platforms [8];
- Ten meters above sea level, which is the most frequently used standard [9].

The latter value is commonly applied in the analysis and design of the ship motion control systems because this distance is smaller or equal to the height of a freeboard of the majority of passenger and cargo vessels [1].

The purpose of a study, the results of which are presented here, was the measurement and modeling of wind parameters affecting manned ship models. These models are used on a small lake for training in shiphandling as well as for research purposes. The results of the bibliographic query indicate that no relevant investigations concerning models of wind influence on such processes have been conducted so far. Existing considerations on wind load on the ship have concerned full-size vessels [3,10,11], while analyses of the nature of the wind blowing over the surface of a lake or similar water areas have referred usually to the wind spatial models or environmental issues [12,13].

The contribution of this research may be relevant because:

- Scale models of ships have been frequently used by numerous research teams worldwide for testing and verification of various marine control systems in recent years, especially in the area of autonomous shipping [14,15,16,17,18,19,20,21,22,23]. One of the key elements in the process of designing the a motion control systems, is not only a reliable mathematical model of a ship dynamics, but also the model of the environment in which she moves. This allows us to simulate, test and verify the developed solutions;
- The wind and wave dynamics model is, as already mentioned, a desirable component in feed-forward disturbances compensation part of control system when a vessel is maneuvering at a low speed in confined waters or is performing DP (Dynamic Positioning) in harsh weather conditions [1,24];
- In the case of ASV (Autonomous Surface Vessel) operating on lakes or water areas of a similar nature, presented results of the investigations can be used directly to some extent, because many of these units are similar in dimensions to the scale models for which the wind and waves induced forces were to be estimated [25].

The specificity of the experiments caused, due to the dimensions of the scale models, the direction and speed of the wind to be measured at a very small altitude above the water surface. Hence, the goal was to check whether and to what extent the obtained results in the form of empirical functions of instantaneous wind speed spectral density are comparable to the models commonly used in ship control systems.

This work had a limited scope. Its goal was not to obtain an accurate, general mathematical model of the wind blowing over the certain type of lakes. Its intention was to obtain a model of wind disturbances suitable for environmental forces simulation for the scale ship motion control system synthesis.

The paper is structured as follows: in Section 2, the mathematical background of wind measurement is explained, particularly the problem of measured signals sampling and filtering. Specific experiment conditions caused by the dimensions of the scale ship are addressed in this part too. Section 3 contains description of the hardware assembled in the project. In Section 4, the mathematical formulation of the selected standard power density functions of wind speed turbulences are presented. They are used as a set of reference points for the analyses of the measurements results, which are detailed in Section 5. Finally, the research outcome is discussed and summarized in Section 6.

## 2. Wind Modeling Principles

These studies about wind modeling and spectra estimation are based on the real acquired data. Wind speed and direction values are transmitted via serial interface and recorded. Therefore, one should take into account digital signal processing limitations. Beyond them are the probability of obtaining a lower gust value than measured one, which should be relatively low to model the turbulent wind spectrum. Power spectral density and turbulence intensity are used to describe wind turbulent flow in the ‘boundary layer’. Moreover, in wind modeling principles described in the subsections below, wind power law describing change of wind speed with height was included.

### 2.1. Sampling and Filtering of Measurement Data

Nowadays almost all wind measurements are digitized or performed using of digital equipment. Sampling frequency selection is a first parameter which should be carefully chosen during system design. It has a direct influence on the measurement quality. Beeljars in [26] proved that probability of obtaining lower value after digitization than measured one is defined by:

(2) $$Pr\left(\frac{U_{max}-\bar{U}}{\sigma }<U_{s}\;,\;T\right)=e^{-\xi },$$

with

(3) $$\xi =\frac{T}{\Delta }\frac{a}{\pi }e^{-\frac{1}{2}{U_{s}}^{2}}\left(1-\frac{1}{3}\left(1+\frac{U_{s}^{2}}{2}\right)a^{2}+\ldots \right)$$

and

(4) $$a={\left(\frac{1-\frac{R(\Delta )}{R(0)}}{1+-\frac{R(\Delta )}{R(0)}}\right)}^{1/2},$$

where *T* is the sampling period, Δ is the interval between samples, $U_{s}$ is the wind speed, $\bar{U}$ is the mean wind speed, σ is the standard deviation and R(Δ) is the covariance between samples.

Equations (2)–(4) are valid for small sampling period: $f_{s}\geq 2$ Hz. It corresponds to lower recorded wind gust speed than real, physical wind speed. It is important to obtain low probability of it during measurement. It was assumed that Pr≤0.1 of the turbulent wind spectrum modeling is acceptable for the purpose of ship control system synthesis.

The output rate of the Gill WindObserver II ultrasonic anemometer may be set to the value ranging from 1 Hz to 10 Hz. The probability of obtaining a lower digital value than the measured one for this device ranges from 0.14 to 0.02, respectively.

As it was stated in (1), wind speed $\tilde{V}\left(z,t\right)$ measured by the anemometer may be divided into two components: mean speed $\bar{U}\left(z\right)$ and turbulent wind speed v(z,t). Contemporary perception of these components describes a mean wind speed as a slow varying factor with fluctuations corresponding to the synoptic scale and turbulent wind speed as an element depending on the local winds [27]. To extract the slow varying part from measurements data preprocessing, smoothing is necessary. It is observed that 10 min averaging gives sufficient information for most applications [26]. In this case, wind speed standard deviation changes slightly and extreme gusts are possible to register.

Since wind measurements gathered for this work are used for future modeling of wind interactions with scale ship in motion, the time related parameters in data processing should be scaled too, according to the Froude laws of similitude [28]. Ship models in scope of this project are built in λ=1:24 scale; therefore, time ought to be scaled by $\lambda_{t}=\sqrt{\lambda }≃5$. Consequently, in this work, averaging in every 120 s was assumed, which also corresponds roughly to the training ships’ time constant $T_{s}≃120$ s. Taking into account the ship’s inertia, the mean value of the external disturbance causing drift and yaw moments should be analyzed for a period corresponding to the time constant, and turbulence should be regarded as force causing temporal change in drift $\Delta F_{y}$ and yaw moment $\Delta N_{r}$.

In digital signal processing, according to the Nyquist theorem, maximal signal frequency should be less or equal to half of the sampling frequency. Therefore, only gusts lasting longer than 200 ms are analyzed when data are sampled with frequency $f_{s}=10$ Hz, which corresponds to $f_{max}=5$ Hz in the digital signal spectrum. It is equal to a wind gust lasting about 10 s acting on the full-scale ship due to wind speed scaling by $\lambda_{t}$. It can be regarded still as fast-changing external disturbance, comparing to the ships inertia and plant time constant.

Beljaars has shown [26] that extreme values are affected by the data filtering process and as the averaging period increases, the signal standard deviation decreases; however, it is necessary to smooth data before turbulent wind spectrum computation, thus moving average filter was applied to the raw data. It was chosen due to its ability to reduce random measurement noise while retaining sharp step response. Due to the impact of the various configurations, from 10 to 200 points, the moving average filter application on the measurement data quality was tested. Finally, filtration over 100 samples was realized.

### 2.2. Applied Methodology of the Wind Measurements

Turbulent wind velocity is analyzed statistically using a power spectrum density (PSD) function, where all loads are analyzed in a frequency domain being compared to the total wind turbulence power. Wind spectrum was then computed using discrete Fourier transform (DFT) according to Formula (5).

(5) $$S\left(n\right)=\frac{|DFT\left(\tilde{V}\left(z,t\right)\right){|}^{2}}{f_{s}N}\;\left[m^{2}/s^{2}\right]$$

where *n* is the frequency, $f_{s}$ is the sampling frequency and *N* is the number of data points used in DFT.

**Figure 1:**
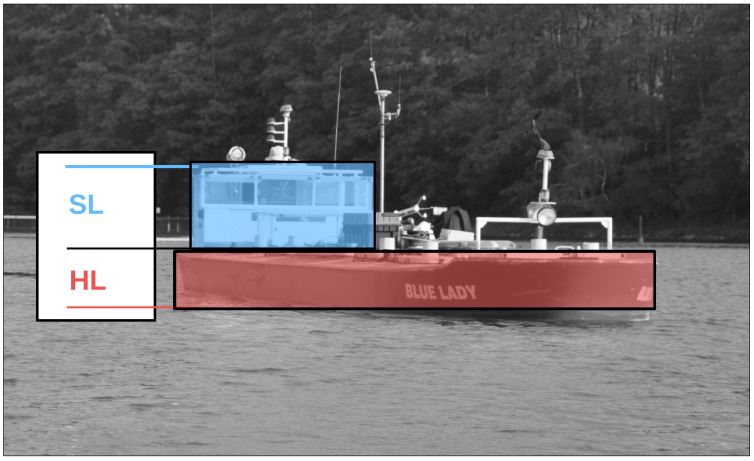
Ship silhouette with marked wind impact areas: HL—hull layer, SL—superstructure layer.

As mentioned earlier, standard wind measurement height for meteorological purposes is equal to 10 m from the ground. For the planned wind model and simulation purposes, it was not applicable due to scale ship dimensions. Her freeboard is about 0.7 m high and her superstructure reaches 1.7 m. Wind acting on the lower and upper ship parts causes mainly drift $\Delta F_{y}$ and yaw moment $\Delta N_{s}$, respectively, hence for the wind forces modeling, the hull and superstructures areas are analyzed separately [11]. In our project, wind measurement heights were reduced to approximately 0.5 m and 1.5 m. Scale ship silhouette with marked hull (HL) and superstructure layer (SL) wind impact areas is presented in Figure 1.

According to the present state of the knowledge, air movement in the ‘boundary layer’ is resisted by the frictional effects of the rough surface. This leads to the turbulent flow, where the level of gustiness, described as turbulence intensity *I*, is defined as [29]:

(6) $$I=\frac{\sigma_{u}}{\bar{U}},$$

where $\sigma_{u}$ is wind speed standard deviation over its mean value $\bar{U}$ usually computed over 10 min intervals. For this research purpose, according to the time scaling law, it was computed for 120 s intervals.

Intensity of turbulence *I* is greater in the uneven terrain [30]. The roughness estimated for the Silm Lake surface $z_{0}$ ranges from single millimeters to approximately 10 cm.

There is a correlation between wind speed and the measurement height. Wind is slowed down along the surface and this phenomenon is called wind shear. It is described by the wind profile power law:

(7) $$\bar{U}\left(z\right)={\bar{U}}_{10}{\left(\frac{z}{z_{10}}\right)}^{\alpha },$$

where α=1/7 is the surface roughness exponent, $\bar{U}\left(z\right)$ is the mean measured wind speed at *z* meters and ${\bar{U}}_{10}$ is the wind speed at $z_{10}=10$ m.

In the presented research Equation (7) was used to compute mean wind speed at 10 m above the lake surface, needed for the standard turbulent wind spectra estimation.

## 3. Measurement Hardware Setup

**Figure 2:**
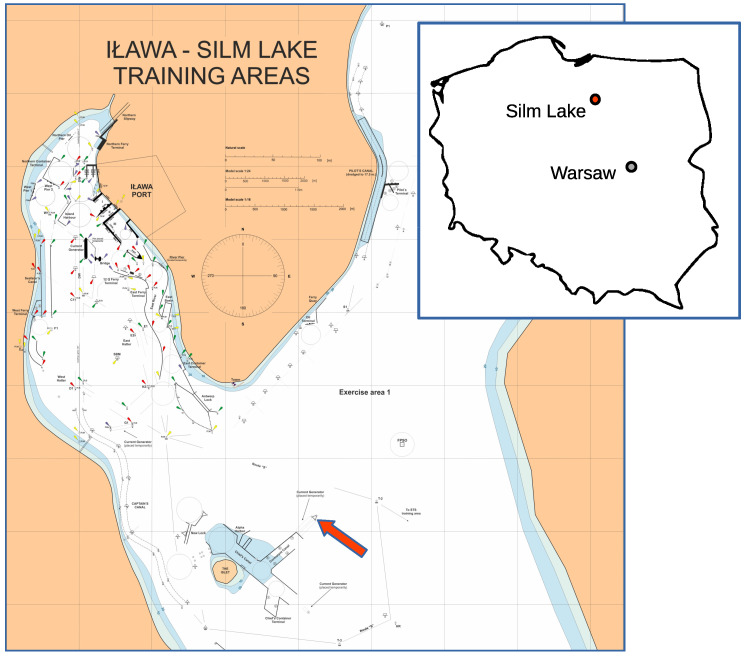
Silm lake map showing measurement hardware mounting place (marked with red arrow) and sketch of Poland borders with approximate location of this lake.

Wind data were collected on the Silm lake, near Iława, Poland. Figure 2 shows the approximate geographical location of it. The lake is a venue of the Shiphandling Research and Training Center where ship manned models are used, mainly for training purposes [31].

**Figure 3:**
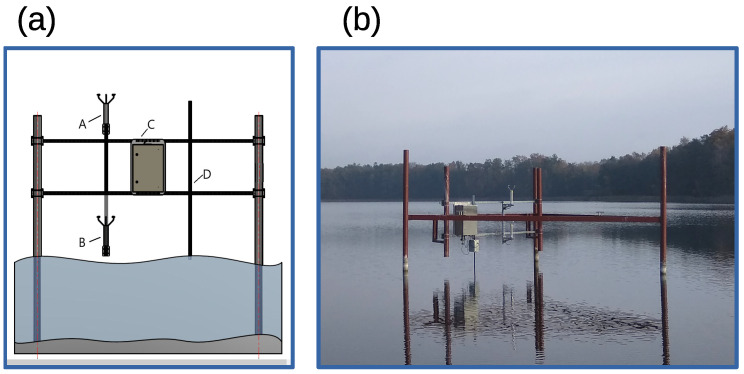
Measurement device: (**a**) design concept: A,B—ultrasonic anemometer, C—measurement box, D—wave sensor; (**b**) device mounted at the test point.

In order to obtain possibly undisturbed data, the measurement device was placed in the lake in dedicated construction as depicted in Figure 3. The measurement system location was chosen to ensure unrestricted wind flow from all possible directions as well as to keep the device out of the main maneuvering areas (see: Figure 2). Despite that, the installation was enclosed in steel barriers to prevent losses caused by accidental collision with maneuvering ship model.

**Figure 4:**
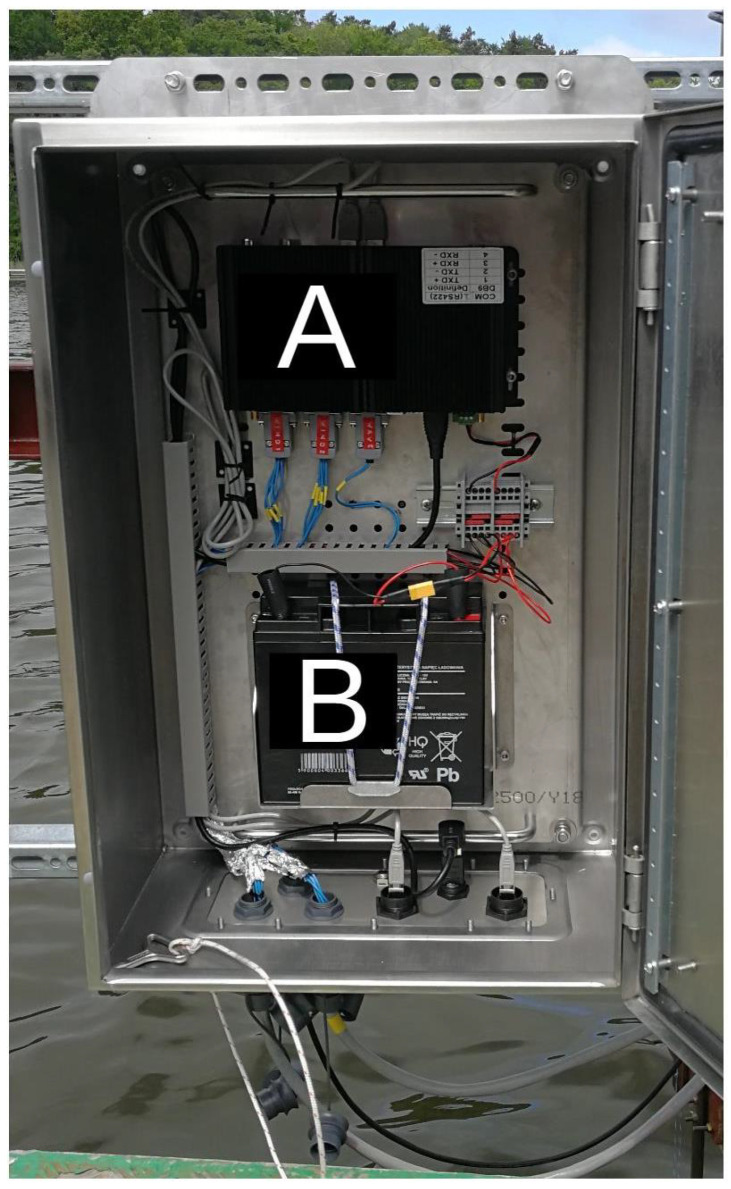
Measurement box arrangement: A—computer executing real-time sampling in the measurement system and data logging; B—12 V, 20 Ah battery.

The measurement system consists of four main components, which are placed on the mechanical structure presented in Figure 3. There are two Gill WindObserver II ultrasonic anemometers (A, B) located one above the other. As explained in the previous section, they are placed 1.5 (device A) and 0.5 (device B) meters above the water surface, respectively. In order to investigate relations between wind parameters and the wave properties the wave sensor (D) was also installed. All data were collected and registered with the sampling rate 10 Hz. A battery-powered real-time target computer, located in the waterproof box (C), was used. This configuration allowed for 12-hours uninterrupted data logging. Arrangement of the box is shown in Figure 4.

**Figure 5:**
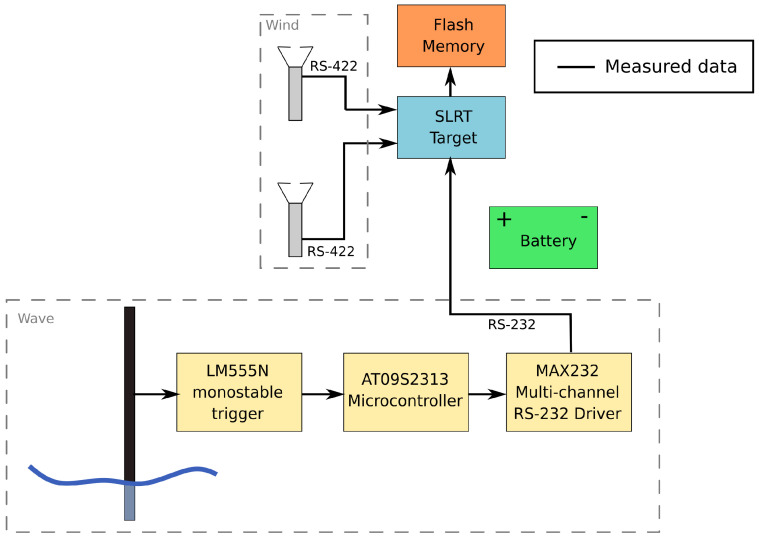
Block diagram of the measurement hardware setup.

Wave height measuring device marked as “D” in Figure 3a was adopted from a previous version of the measurement system described in [32]. It involves sensor build as a capacitor whose electrodes are: flat bar made of bronze and copper wire in a Teflon sheath, timer, microcontroller and RS-232 driver. Data logging software with 10 Hz sampling rate was implemented using Simulink Real-Time Target toolbox from the MATLAB-Simulink environment. Data from anemometers are logged directly. Data logging from wave meter requires the LM555 timer combined with microcontroller and multichannel RS-232 driver application. Block diagram of the complete system, with indicated parts responsible for wave and wind measurements, is presented in Figure 5.

Hardware setup presented in this section was used in all executed experiments. Measurements were carried out in several 12-h sessions in summer months from May 2020 to August 2022. The site is not accessible, depending on the local weather, from late October to early April.

## 4. Standard Turbulent Wind Spectra

One of the most relevant goals of this research was to check whether the standard turbulent wind spectra are suitable to describe character of wind measured in the very low level above lake surface. The main issue was to find the spectrum that was either scaled or non-scaled and was able to represent the measured wind frequency characteristic with acceptable accuracy.

There exist numerous formulations of power density spectra describing wind gustiness. Almost all of them have been defined in the second half of the 20th century. Hence, the problem of the air turbulent flow description in the frequency domain is not a new one. Due to the frequent use in description of wind impact on full-size ships, we decided to compare the empirical PSD of measured signals with the ones from following formulas: non-normalized von Karman (1948), Davenport (1961), Harris (1968), Kaimal (1972) and Eurocode (1991).

The von Karman mathematical model for continuous gusts [33] describes wind power density with the use of Bessel functions with an imaginary argument. This parameterized model was presented in NACA (National Advisory Committee for Aeronautics) Report [34] in 1957, in a present form. It was designed for aviation-related applications. Therefore, in the original research, it was formulated for three linear components (*u*—longitudinal, *v*—transversal and *w*—vertical). For the purpose of ship external disturbances modeling and simulation, authors take into consideration only longitudinal wind PSD given as:

(8) $$nS_{u}\left(n\right)=\frac{47x_{u}^{2}\sigma_{u}^{2}}{{\left(1+70.8x_{u}^{2}\right)}^{\left(5/6\right)}},$$

where *n* is the frequency, *z* is the measurement height, $\sigma_{u}$ is the wind speed standard deviation and *x* is computed according to formula:

(9) $$x_{u}=\frac{nL_{u}}{\tilde{V}\left(z\right)}.$$

The von Karman turbulence model is parameterized by the roughness length $z_{0}$ dependent on the terrain profile and integral scale of turbulence denoted $L_{u}$. The authors decided to take into consideration two separate formulas for integral scale of turbulence computation from Couniham, used by Solari [35], denoted $L_{C}$:

(10) $$L_{C}=300\;{\left(\frac{z}{300}\right)}^{0.46+0.074\ln z}$$

and the new one proposed by ESDU [36], denoted as $L_{ESDU}$

(11) $$L_{ESDU}=25\;z^{0.35}\;z_{0}^{-0.063},$$

where *z* is the wind measurement height.

In the present form, with integral scale of turbulence proposed by ESDU, it is one of the most popular turbulence models in aircraft design due to possibility of stochastic output process computation on the basis of filters accepting white noise as input. This model, compliant with MIL-F-8785C Specification, is also implemented in Aerospace Toolbox in MATLAB, what indicates its wide acceptance.

Davenport proposed in [37] spectral model of strong winds, which is described by the equation:

(12) $$nS_{u}\left(n\right)=\frac{0.667x^{2}\sigma_{u}^{2}}{{\left(1+x^{2}\right)}^{\left(4/3\right)}},$$

where *n* is the frequency, *z* is the measurement height, $\sigma_{u}$ is the wind speed standard deviation and *x*, which is computed according to

(13) $$x=\frac{nL}{\tilde{V}\left(z\right)},$$

where *L* is the integral length scale of turbulence, which for Davenport spectrum is equal to L=1200. It is considered as a mean spectrum for measurement height 10 m <z<150 m now. Authors decided to compare own measurement results to this standard spectrum, because in a original form it was defined as turbulent spectrum for strong winds near the ground [37].

Harris and Kaimal wind spectra have been commonly used since 1980s for modeling of the wind loads acting on the offshore constructions. They both are compared in the literature with real measurements data and used for loads computation. Moreover, International Electrotechnical Commission in IEC-61400-1 Standard [38] recommends Kaimal model use for the wind turbulence modeling in wind turbines issues. Consequently, taking Kaimal spectrum into consideration is also reasonable. The intent of studies presented in this paper was to provide model of loads on the ship due to wind gusts. Therefore, both spectra were taken into consideration during comparative analysis of measurements.

Harris presented formula for the spectrum computation in [39] as a gustiness in high winds in a form:

(14) $$nS_{u}\left(n\right)=\frac{3}{5}\frac{x\sigma_{u}^{2}}{{\left(2+x^{2}\right)}^{\left(5/6\right)}}$$

with

(15) $$x=\frac{nL}{V_{10}},$$

where L=1800 and $V_{10}$ is wind speed reduced to the standard measurement height $z_{0}=10$ m.

Kaimal spectrum was published in [40] and described by relation:

(16) $$nS_{u}\left(n\right)=\frac{100x\sigma_{u}^{2}}{3{\left(1+50x\right)}^{\left(5/3\right)}}$$

with

(17) $$x=\frac{nz}{\bar{U}}.$$

Eurocode wind spectrum was proposed as modified Solari spectrum in European Standard EN 1991-1-4 [41]. It was designed for wind loads on the structures computation and is widely used in civil engineering and architecture. Due to its popularity, the authors decided to check if its application in wind modeling for the marine purposes is reasonable. Eurocode wind model is given by:

(18) $$nS_{u}\left(n\right)=\frac{6.8x\sigma_{u}^{2}}{{\left(1+10.2x\right)}^{\left(5/3\right)}}$$

where $L_{C}$ is given by (10) and corresponds to the Solari integral scale of turbulence.

All above-mentioned wind spectrum models are empirical ones, based on the wind power law. Nowadays, all of them are calculated numerically, and their parameters are sensitive to the cut-off frequencies [42]. Standardization leads to the parametrization of turbulence integral scale length, as required by the dedicated European and US institutions. One may obtain insight into these issues through published standards (i.a., ESDU, Eurocode, NACA).

## 5. Results

**Figure 6:**
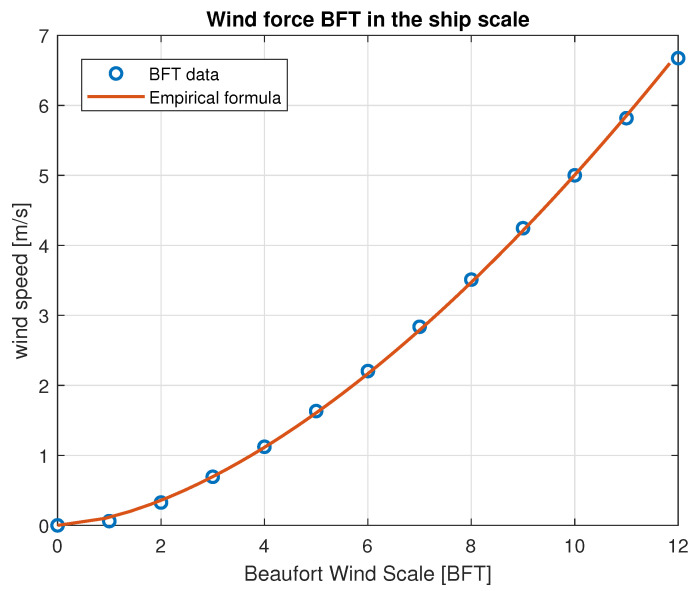
Conversion of wind speed to the BFT (Beaufort Wind Scale) in the ship model scale.

Measurement data analyzed in this section were collected using hardware described in Section 3 according to the rules presented in Section 2. For further investigations, only several chunks of whole sets of data were chosen. The principle behind the selection was to reduce the amount of processed data, but without losing information about the characteristics of the wind for its different speeds and directions. Data examination was divided into three stages: presentation of signal time histories, basic statistical processing and spectral analysis. Each of the stages are discussed in this section individually. Moreover, because our findings are to be used for disturbances modeling of the ship automatic control system, they were recalculated to the wind force in Beaufort scale fitted to the scale of the ship model. These rescaled values were used merely to make the displayed figures more understandable. For the other calculations, natural, physical values were used. The speed recalculation principle is presented in Figure 6.

According to the conversion presented above four wind conditions determined by different wind speed ranges were analyzed. These ranges are presented in Table 1 set a framework for the measurement results presentation.

**Figure 7:**
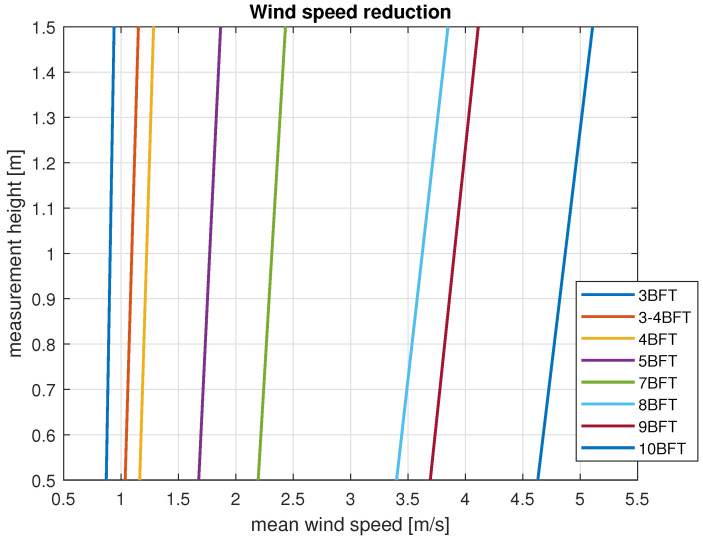
Wind speed reduction for all registered wind forces in BFT in ship scale.

As we noted in previous sections, wind speed and direction were recorded for the two heights above the water surface. The results of the average wind speed in different conditions are presented in Figure 7.

Analysis of this data shows that wind speed for all registered categories decreases with the measurement height reduction. In order to prove measurements quality and to validate data, compliance of the obtained results with the wind profile power law was checked. Analysis was performed based on the high anemometer mean wind speed for 120 s periods. According to the Equation (7) mean wind speed at the 0.5 m above the water level was estimated. Results were compared with mean wind speed registered by the low anemometer and collected in Table 2.

(19) $$\bar{U}\left(z_{1}\right)={\bar{U}}_{z_{2}}{\left(\frac{z_{1}}{z_{2}}\right)}^{1/7}$$

where $\bar{U}\left(z_{1}\right)$ is the mean estimated wind speed at z=0.5 meters and ${\bar{U}}_{z_{2}}$ is the measured wind speed at $z_{2}=1.5$ m.

Achieved results show that the consistency of measurements and calculations reached an average level of 5% of the measured value. It demonstrates that gathered data are suitable for the future analysis and wind profile power law is preserved.

### 5.1. Measurements

**Figure 8:**
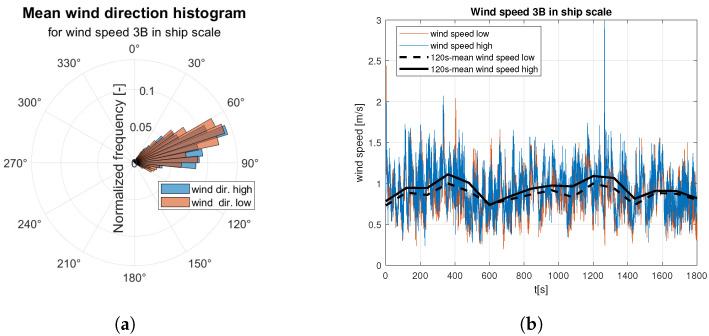
Raw measurements for weak wind—3BFT in ship scale: (**a**) wind rose; (**b**) time series.

**Figure 9:**
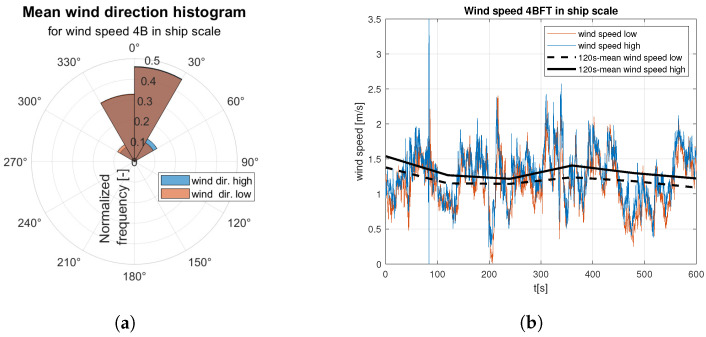
Raw measurements for weak wind—4BFT in ship scale: (**a**) wind rose; (**b**) time series.

**Figure 10:**
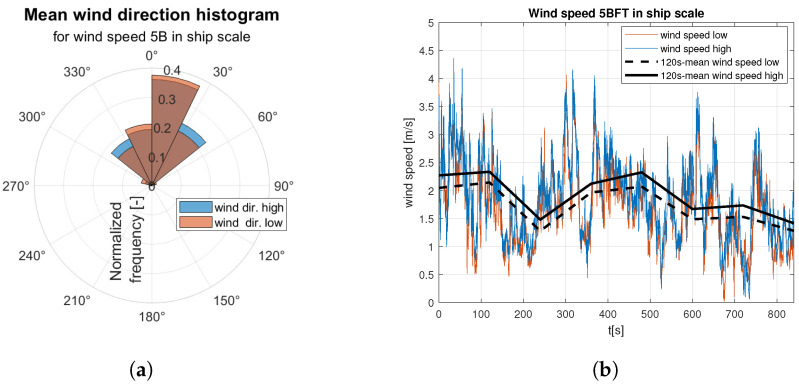
Raw measurements for medium weak wind—5BFT in ship scale: (**a**) wind rose; (**b**) time series.

**Figure 11:**
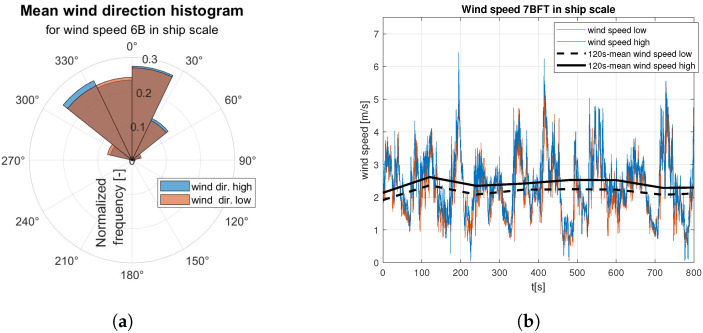
Raw measurements for medium weak wind—6BFT in ship scale: (**a**) wind rose; (**b**) time series.

**Figure 12:**
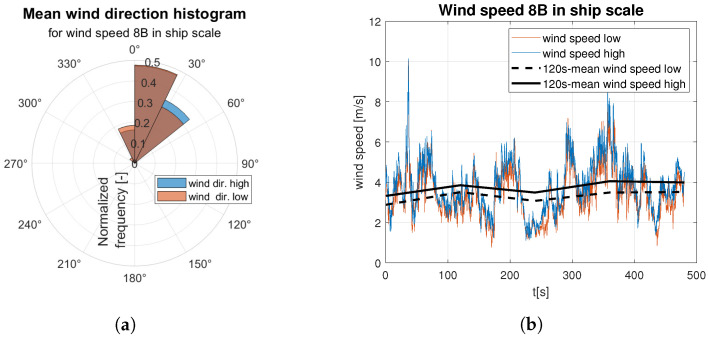
Raw measurements for medium strong wind—8BFT in ship scale: (**a**) wind rose; (**b**) time series.

**Figure 13:**
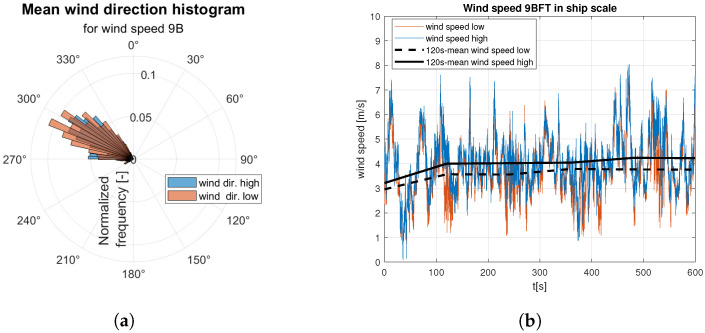
Raw measurements for strong wind—9BFT in ship scale: (**a**) wind rose; (**b**) time series.

**Figure 14:**
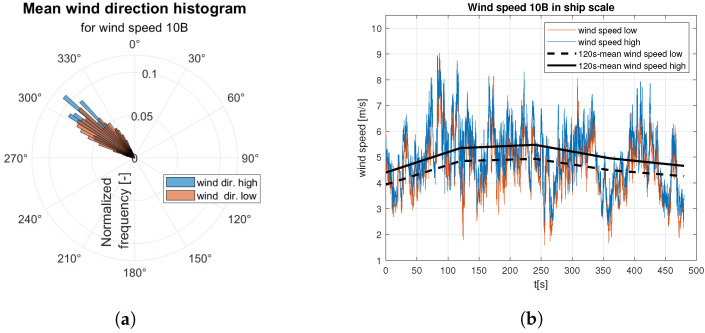
Raw measurements for strong wind—10BFT in ship scale: (**a**) wind rose; (**b**) time series.

Raw data are divided into two sets of anemometer outcome of wind speed and direction registered about 0.5 m and 1.5 m above water surface. They are presented as wind rose and wind speed time domain graph. Raw data are indicated by the red and blue lines according to the colors marked the “hull layer” (HL) and “superstructure layer” (SL) in Figure 1, respectively. It corresponds with “wind low” and “wind high” labels in Figure 8, Figure 9, Figure 10, Figure 11, Figure 12, Figure 13 and Figure 14. Mean values of 120-s time spans are indicated by black solid and dashed lines for upper and lower anemometer measurements, respectively.

Figure 8 and Figure 9 illustrate raw measurements of the weak wind. In mean wind direction histogram for 3B (Figure 8a) in ship scale a much smaller data dispersion is observed than for 4B in ship scale (Figure 9a).

Figure 10 and Figure 11 illustrate registered wind directions and speeds for the medium weak wind category. On 5B in ship scale wind rose (Figure 10a) main wind direction is observed and gusts from the other directions appear twice as often. On the Figure 11a a distribution of gust directions close to uniform is observed. Moreover, for the stronger winds from the medium weak category the average wind speed is close to constant (Figure 11b), in contrast to oscillating mean value presented in Figure 10b.

In case of medium strong (Figure 12) and strong (Figure 13 and Figure 14) wind categories an increase in dispersion of the gust directions is observed with the wind speed increase (Figure 12a, Figure 13a and Figure 14a). In both categories mean wind speed value remains almost constant, which is shown in Figure 12b, Figure 13b and Figure 14b.

### 5.2. Statistical Data Analysis

**Figure 15:**
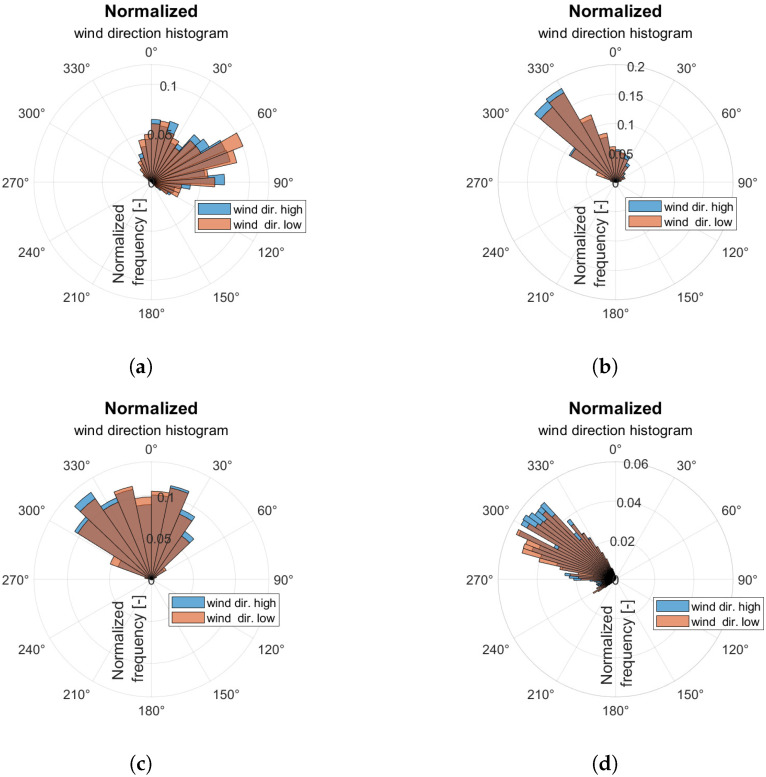
Normalized wind direction histograms for 2.5 h experiments: (**a**) week wind; (**b**) medium week wind; (**c**) medium strong wind; (**d**) strong wind.

**Figure 16:**
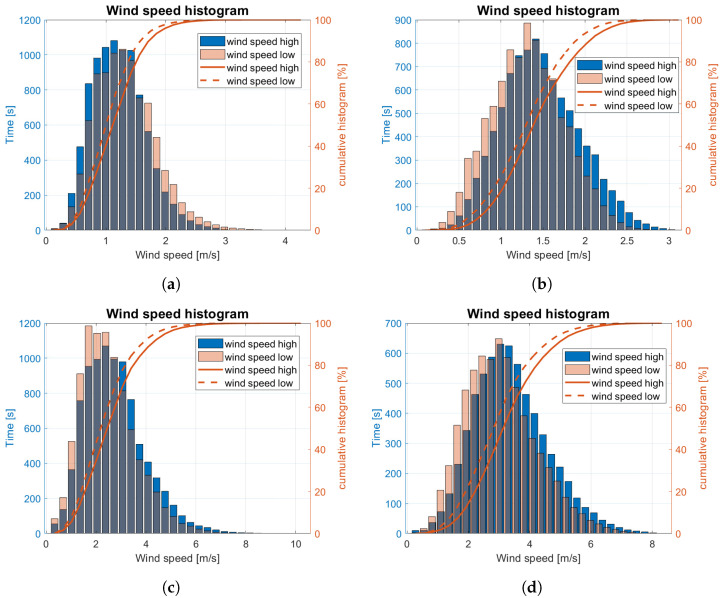
Normalized wind speed histograms for 2.5 h experiment: (**a**) week wind; (**b**) medium week wind; (**c**) medium strong wind; (**d**) strong wind.

In this stage, the normalized wind direction histograms for all four wind categories (Table 1) were prepared. They are displayed in Figure 15. Similarly, wind speed histograms for the same data were estimated end depicted in Figure 16. Each of the diagrams is based on processed data samples of 2.5 h measurement for each wind category.

In weak and medium strong wind conditions, significant deviations of gusts from the average wind direction are observed. Deviation of wind direction extends to 90°. Moreover, in case of medium-strong wind (Figure 15c) its direction histogram is close to an even distribution. In other wind conditions (Figure 15a,b), the dominant wind direction occurrence is observed. Furthermore, in the strong wind regime, near the water level, an even distribution on two wind directions appears. In contrast, it is not clearly visible for the higher anemometer measurements.

In Figure 16, wind speed histograms are compared with the cumulative histograms for four wind categories. Curves similar to the shape of the Gaussian function have been obtained for medium weak and strong winds (Figure 16b,d). In other cases, a shift of the maximum value towards lower velocities is observed (Figure 16a,c).

Wind speed standard deviations and wind gust intensity are collected in Table 3 for both anemometer mounting heights. Wind speed standard deviation increases with the wind speed increase for both measurement heights. In contrast, the biggest gust intensity is observed in medium weak wind conditions (5–6 BFT in ship scale). It correlates with slight variations in wind direction presented in Figure 15b.

### 5.3. Measured Wind Spectra

**Figure 17:**
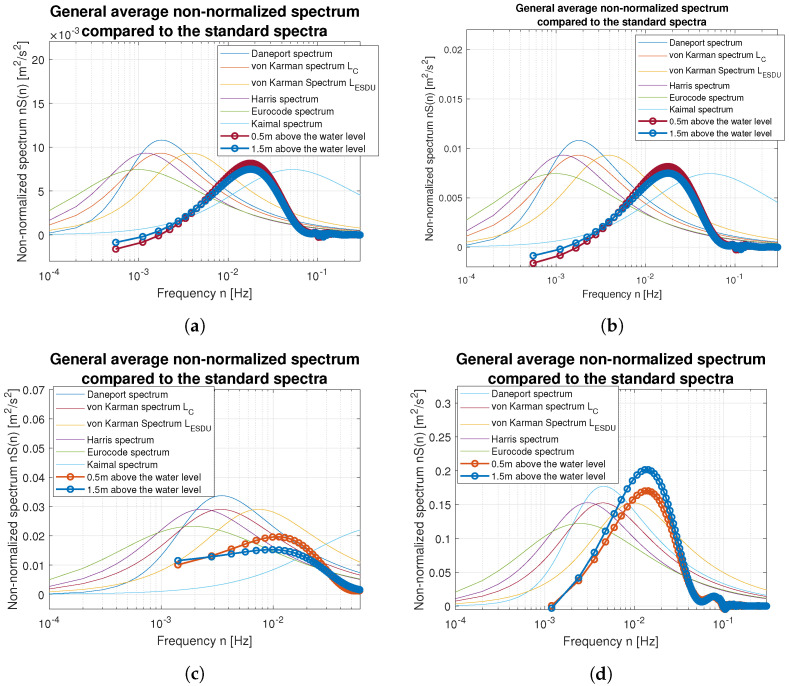
Estimated spectra compared to standard ones: (**a**) Weak wind (3 BFT in ship scale); (**b**) Weak wind (4 BFT in ship scale); (**c**) Medium weak wind (5 BFT in ship scale); (**d**) Medium strong wind (6 BFT in ship scale).

**Figure 18:**
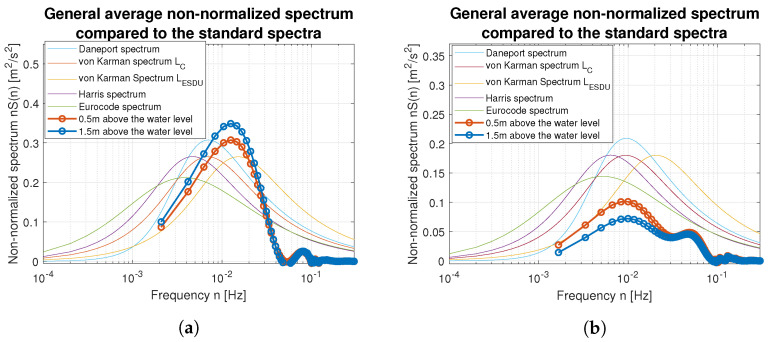
Estimated spectra compared to standard ones: (**a**) Strong wind (8 BFT in ship scale); (**b**) Strong wind (10 BFT in ship scale).

Smoothed wind measurements were used to compute their power spectral density (PSD) functions and to compare them with standard spectra widely used in the area of wind modeling. Figure 17 and Figure 18 show how PSD functions estimated from measurements gathered for different wind speed are similar to the standard ones. It was decided to present non-normalized spectra for better comparison and to show all possible similarities and differences in the frequency domain. The graphs are prepared in log-line reference frames.

Simple comparison of curves plotted in Figure 17 and Figure 18 allow us to point the standard spectrum closest to the estimated from lake measurements for each of the analyzed cases. They are plotted separately in Figure 19, Figure 20, Figure 21, Figure 22, Figure 23 and Figure 24.

Quantitative analysis of the computed PSD and standard spectra for each wind force is presented in Table 4. Mean squared error (MSE) was computed as a sum of squared differences between PSD model and PSD computed on the basis of experimental data. Presented in Table 4 MSEs are the best fits to standard spectra, named in the third column. Acceptable fit at 0.05 level was obtained only for two spectra—for wind force 3 and 5 BFT in ship scale. Usage of the other standard spectral models will need their empirical scaling.

## 6. Discussion

The results of the experiments allow us to conclude that the phenomena concerning wind speed and direction in the thin boundary layer just above the lake water level are similar in nature to analogous phenomena studied for layers of much larger size. Changes of the wind speed in this thin layer can be modeled by aggregation a constant or slow-varying mean value and turbulence which is a stochastic process using similar methods of synthesis to those previously used in full-scale case. The measurements processing also revealed the wind shear phenomenon. Unfortunately, velocities were measured only for two heights above the water level. However, to verify correctness of the presented measurement method and to prove that wind profile power law has been preserved wind speed at 0.5 m was estimated. Obtained results show that the agreement of measured and estimated wind speeds at level 5–7% was obtained. This result suggests that the recorded reduction in wind speed appeared to follow the wind profile power law.

The study was naturally limited in scope. Winds were not measured during the autumn and winter seasons, as the research center is inaccessible then, nor were winds analyzed at speeds that, when converted to the model scale, would exceed those naturally occurring in the atmosphere over the marine areas.

With the results obtained in the form of a power spectral density function of the longitudinal velocity component of turbulence, a gap can be filled in modeling the effects of wind for small craft moving over a lake or similar water area. So far, this has been performed using models that were not designed for vessels whose freeboard height most often does not exceed 1 m. Scale models of ships tested in open waters are increasingly used as a tool for design of automatic control systems of ship motion. This is clearly visible in the area of research on autonomous ships. Thus, a more accurate wind disturbances model will be a useful tool to improve the quality of ongoing work, especially in the subsystems of simulation and verification of control systems.

The obtained forms of the power spectral density function for winds measured for different average speeds indicate that, as in the case of full-scale measurements, it is not possible to identify a single relationship that would be a representation of the phenomena for the full range of speeds. It was shown that for low wind speeds, corresponding to 3–4 BFT in the model scale, the obtained function corresponds to the Kaimal spectrum; for medium speeds: 5–6 BFT in the model scale, to the Von Karman spectrum; for high speeds corresponding to 8 BFT to the PSD function defined by Devenport; for very strong winds, 10 BFT in the model scale corresponds to the Eurocode spectrum.

Thus, at the current stage of research, the best form of modeling wind disturbances for free-running scale ships seems to be a multi-element structure, in which, depending on the wind mean speed, sub-components corresponding to the above-mentioned standard models of the turbulence power spectral density function are activated.

## 7. Conclusions

In the literature, there is a lack of wind models applicable for manned scale training ships and small unmanned surface vehicles (USV). The uniqueness of these models is related to the wind measurement heights. In order to obtain an acceptable mathematical wind model for simulation and control purposes, wind speeds and directions should be measured, respectively, at the vessel’s freeboard and superstructure height. In fact, measurements should be taken at several centimeters to meters above the water level. For that reason, the wind measurement methodology combined with the hardware setup was developed and presented. Obtained results prove that setup seems to be adequate for this task and that it is possible to create a wind model for special purposes based on the acquired data.

Wind speed statistical analysis shows that wind model for the future control purpose should consist of two parts generating, respectively, mean wind and gusts of the specified intensity. Obtained results show that gusts are more intensive closer to the water surface. In contrast, the increase in the mean wind speed with the measurement height is observed. Gusts direction is independent of the measurement height. Presented analysis results show what the model structure should look like.

Beyond the effective wind modeling methods, there is a use of the digital filter reproducing measured spectrum. Presented results show that there is no possibility to obtain good quality model of turbulence based on one of the standard wind spectra presented in the literature. There are large discrepancies between standard spectra and PSD computed based on the acquired data. Depending on the wind strength, the best fit is obtained separately with the Kaimal, von Karman, Davenport and Eurocode spectra. Unfortunately its not a perfect match. Therefore, the future model will be based on the scaled composition. It is predicted to scale spectra and compute their weighted sum in order to obtain full wind turbulence model for the future control purpose. Having this spectral wind model there will be possibility to design digital reproduction filter acting as a wind gusts source in the time domain.

## Figures and Tables

**Figure 19:**
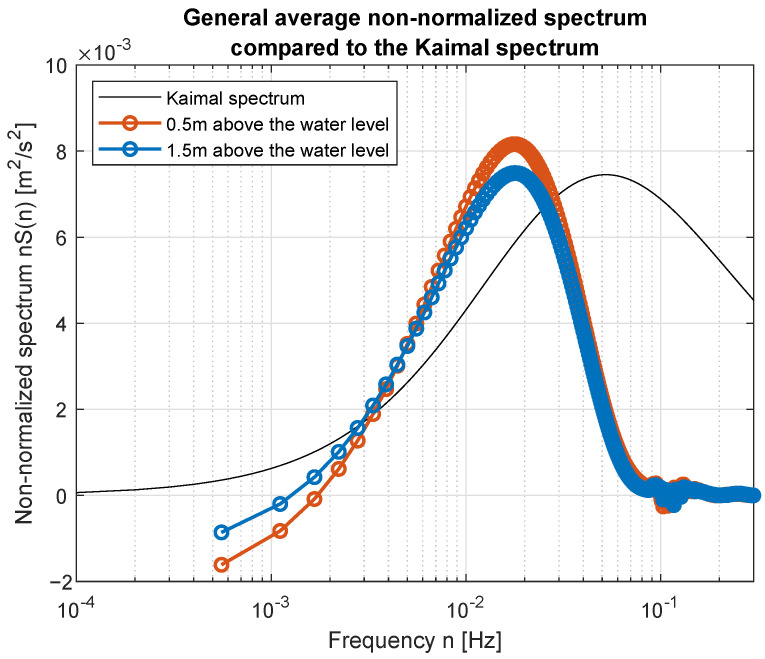
Weak wind (3 BFT in ship scale) compared to Kaimal spectrum.

**Figure 20:**
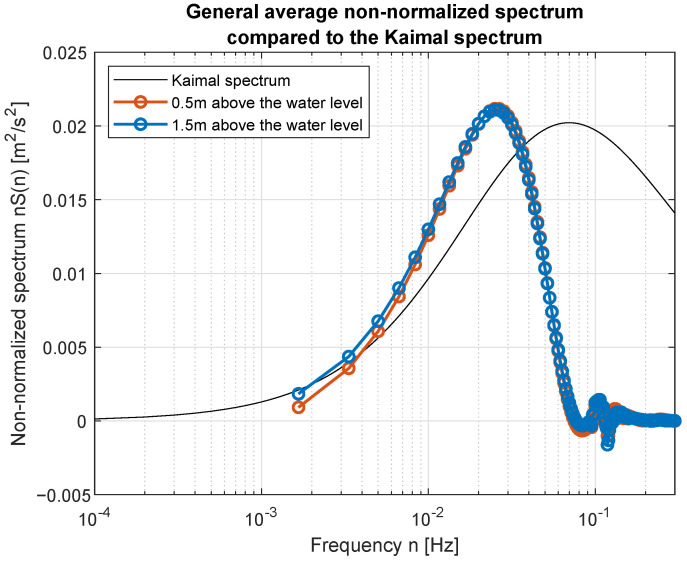
Weak wind (4 BFT in ship scale) compared to Kaimal spectrum.

**Figure 21:**
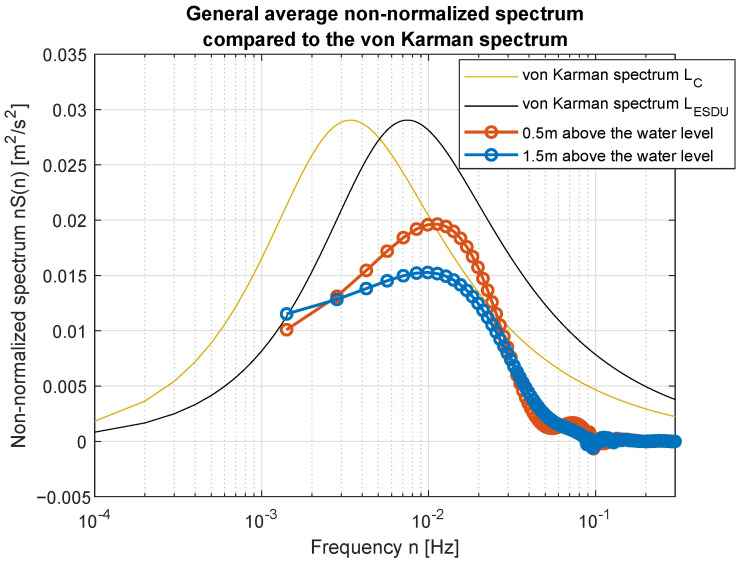
Medium-weak wind (5 BFT in ship scale) compared to Karman spectrum.

**Figure 22:**
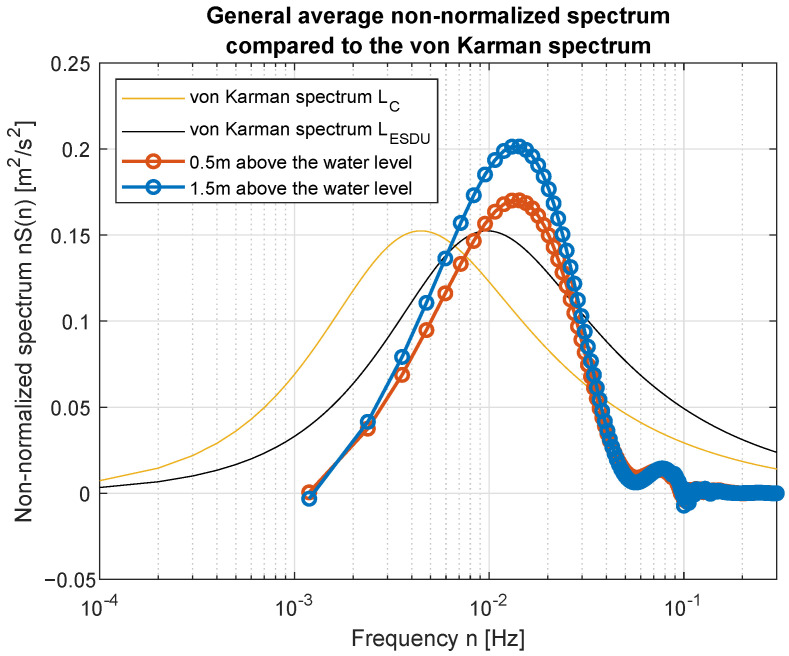
Medium-strong wind (6 BFT in ship scale) compared to Karman spectrum.

**Figure 23:**
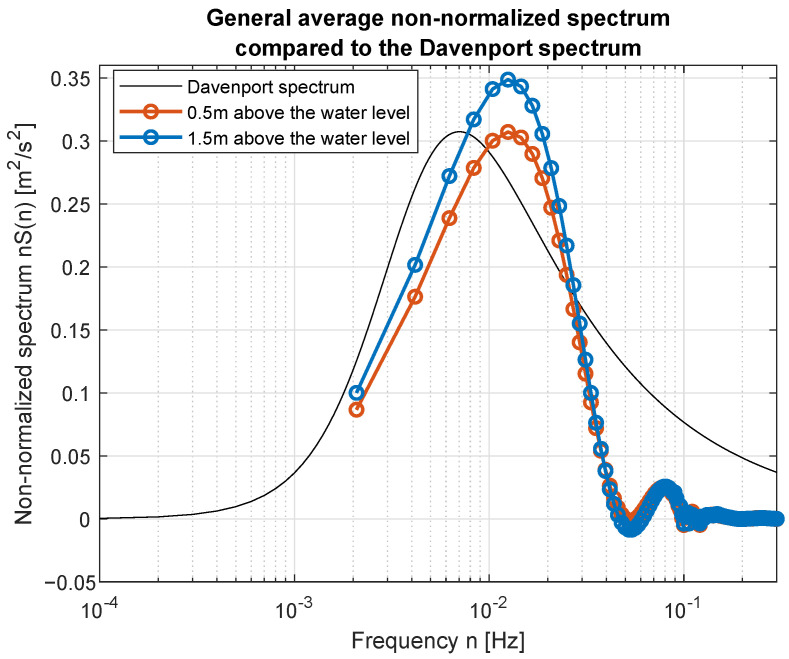
Strong wind (8 BFT in ship scale) compared to Davenport spectrum.

**Figure 24:**
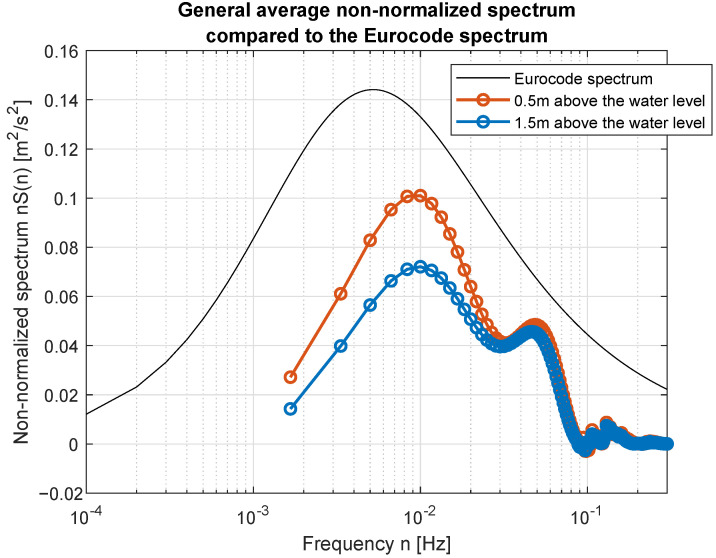
Strong wind (10 BFT in ship scale) compared to Eurocode spectrum.

**Table 1:** Analyzed categories of wind speed.

Wind Category	Wind Speed Range [m/s]	BFT in Ship Scale [BFT]
Weak	0.5–1.0	2–4
Medium weak	1.1–2.2	4–6
Medium strong	2.3–3.5	6–8
Strong	3.6–5.5	8–10

**Table 2:** Verification of the wind profile relationship.

BFT Wind Force in Ship Scale [BFT]	Measured Wind Speed at z=1.5 [m]	Measured Wind Speed at z=0.5 [m]	Estimated Wind Speed at z=0.5 [m]	Estimated and Measured Value Deviation [%]
3	0.94	0.87	0.80	7.9
3–4	1.15	1.04	0.98	5.0
4	1.28	1.16	1.10	5.6
5	1.87	1.68	1.60	4.8
7	2.43	2.19	2.08	5.3
8	3.85	3.40	3.29	3.3
9	4.11	3.69	3.51	4.9
10	5.11	4.63	4.37	5.7

**Table 3:** Statistical parameters of wind sped measurements.

BFT	Mean Wind Speed $\bar{V}$	Wind Speed Standard Deviation $\sigma_{v}$	Gust Intensity *I*	Mean Wind Speed $\bar{V}$	Wind Speed Standard Deviation $\sigma_{v}$	Gust Intensity *I*
		z=0.5 m			z=1.5 m	
3	0.87	0.24	0.28	0.94	0.25	0.27
4	1.16	0.37	0.32	1.28	0.37	0.29
5	1.70	0.68	0.41	1.90	0.72	0.39
6	2.20	0.89	0.40	2.40	0.93	0.38
8	3.40	1.21	0.36	3.80	1.27	0.33
9	3.70	1.14	0.31	4.10	1.12	0.27
10	4.60	1.13	0.24	5.10	1.16	0.23

**Table 4:** Quantitative analysis of data fit to standard spectrum.

BFT Wind Force in Ship Scale [BFT]	Mean Squared Error (MSE)	Corresponding Spectrum
3	0.041	Kaimal
4	0.131	Kaimal
5	0.015	von Karman
6	0.611	von Karman
8	0.842	Davenport
10	0.366	Eurocode

## Data Availability

Data available on request due to restrictions eg privacy or ethical.

## Abbreviations

The following abbreviations are used in this manuscript:

ASV	Autonomous Surface Vehicle
BFT	Beaufort Wind Scale
DFT	Discrete Fourier Transform
DSP	Dynamic Ship Positioning
ESDU	Engineering Sciences Data Unit
IEC	International Electrotechnical Commission
NACA	National Advisory Committee for Aeronautics
PSD	Power Spectral Density

## References

[1] Fossen T.I. (2021). Handbook of Marine Craft Hydrodynamics and Motion Control.

[2] van Berlekom W., Tragardh P., Dellhag A. (1975). Large Tankers—Wind Coefficients and Speed Loss Due to Wind and Sea. Nav. Archit..

[3] Isherwood R.M. (1972). Wind Resistance of Merchant Ships. Trans. R. Inst. Nav. Archit..

[4] Paulauskas V., Paulauskas D., Wijffels J. (2009). Ship safety in open ports. Transport.

[5] Szymonski M. (2019). Some Effects of Wind on Ship’s Manoeuvrability. TransNav Int. J. Mar. Navig. Saf. Sea Transp..

[6] Yi W., Lu Z., Hao J., Zhang X., Chen Y., Huang Z. (2021). A Spectrum Correction Method Based on Optimizing Turbulence Intensity. Appl. Sci..

[7] Knight J.M., Obhrai C. (2019). The Influence of an Unstable Turbulent Wind Spectrum on the Loads and Motions on Floating Offshore Wind Turbines. IOP Conference Series: Materials Science and Engineering.

[8] Forristall G.Z. Wind Spectra and Gust Factors over Water. Proceedings of the OTC Offshore Technology Conference.

[9] Bojórquez E., Payán-Serrano O., Reyes-Salazar A., Pozos A. (2017). Comparison of spectral density models to simulate wind records. KSCE J. Civ. Eng..

[10] Blendermann W. (1994). Parameter identification of wind loads on ships. J. Wind. Eng. Ind. Aerodyn..

[11] Fujiwara T., Ueno M., Nimura T. (1998). Estimation of Wind Forces and Moments acting on Ships. J. Soc. Nav. Archit. Jpn..

[12] Allan J.C., Kirk R.M. (2000). Wind wave characteristics at Lake Dunstan, South Island, New Zealand. N. Z. J. Mar. Freshw. Res..

[13] Krüger O., Schrödinger C., Lengwinat A., Paschereit C.O. Numerical Modeling and Validation of the Wind Flow Over the Lake Wannsee. Proceedings of the 6th European Conference on Computational Fluid Dynamics.

[14] Perera L., Moreira L., Santos F., Ferrari V., Sutulo S., Soares C.G. (2012). A Navigation and Control Platform for Real-Time Manoeuvring of Autonomous Ship Models. IFAC Proc. Vol..

[15] Gierusz W., Łebkowski A. (2012). The researching ship “Gdynia”. Pol. Marit. Res..

[16] Jiao J., Ren H., Sun S., Liu N., Li H., Adenya C.A. (2016). A state-of-the-art large scale model testing technique for ship hydrodynamics at sea. Ocean. Eng..

[17] Hajizadeh S., Seif M., Mehdigholi H. (2016). Determination of ship maneuvering hydrodynamic coeffcients using system identification technique based on free-running model test. Sci. Iran..

[18] Tomera M. (2017). Hybrid Switching Controller Design for the Maneuvering and Transit of a Training Ship. Int. J. Appl. Math. Comput. Sci..

[19] Alfheim H.L., Muggerud K., Breivik M., Brekke E.F., Eide E., Øystein Engelhardtsen (2018). Development of a Dynamic Positioning System for the ReVolt Model Ship. IFAC-PapersOnLine.

[20] Bassam A.M., Phillips A.B., Turnock S.R., Wilson P.A. (2019). Experimental testing and simulations of an autonomous, self-propulsion and self-measuring tanker ship model. Ocean. Eng..

[21] Szelangiewicz T., Żelazny K., Antosik A., Szelangiewicz M. (2021). Application of Measurement Sensors and Navigation Devices in Experimental Research of the Computer System for the Control of an Unmanned Ship Model. Sensors.

[22] Rybczak M., Gierusz W. (2022). Maritime Autonomous Surface Ships in Use with LMI and Overriding Trajectory Controller. Appl. Sci..

[23] Pietrzykowski Z., Wołejsza P., Nozdrzykowski Ł., Borkowski P., Banaś P., Magaj J., Chomski J., Mąka M., Mielniczuk S., Pańka A. (2022). The autonomous navigation system of a sea-going vessel. Ocean. Eng..

[24] Balchen J.G., Jenssen N.A., Mathisen E., Sælid S. (1980). A Dynamic Positioning System Based on Kalman Filtering and Optimal Control. Model. Identif. Control.

[25] Sarda E.I., Qu H., Bertaska I.R., von Ellenrieder K.D. (2016). Station-keeping control of an unmanned surface vehicle exposed to current and wind disturbances. Ocean. Eng..

[26] Beljaars A.C.M. (1987). The Influence of Sampling and Filtering on Measured Wind Gusts. J. Atmos. Ocean. Technol..

[27] Ren G., Liu J., Wan J., Li F., Guo Y., Yu D. (2018). The analysis of turbulence intensity based on wind speed data in onshore wind farms. Renew. Energy.

[28] Dudziak J. (2008). Theory of Ship.

[29] Arany L., Bhattacharya S., Macdonald J., Hogan S.J. (2015). Simplified critical mudline bending moment spectra of offshore wind turbine support structures. Wind Energy.

[30] Klaver E., International Council for Building Research, Studies and Documentation. Working Commission W81 (1996). Actions on Structures: Wind Loads.

[31] Shiphandling Research and Trainig Centre. http://www.ilawashiphandling.com.pl.

[32] Sikora P., Sokół R., Morawski L., Pomirski J. (2008). Wind and wave measurement system for trails on ship models. Pomiary Autom. Kontrola.

[33] Von Karman T. (1948). Progress in the statistical theory of turbulence. Proc. Natl. Acad. Sci. USA.

[34] Diederich F.W., Drischler J.A. (1957). Effect of Spanwise Variations in Gust Intensity on the Lift due to Atmospheric Turbulence. Technical Report. https://ntrs.nasa.gov/api/citations/19930084862/downloads/19930084862.pdf.

[35] Solari G. (1988). Equivalent wind spectrum technique: Theory and applications. J. Struct. Eng..

[36] ESDU (1993). ESDU 86035 Integral Length Scales of Turbulence over Flat Terrain with Roughness Changes.

[37] Davenport A.G. (1961). The Application of Statistical Concepts to the Wind Loading of Structures. Proc. Inst. Civ. Eng..

[38] IEC (2019). Wind Energy Generation Systems—Part 1: Design requirements.

[39] Harris R. (1968). On the Spectrum and Auto-Correlation Function of Gustiness in High Winds.

[40] Kaimal J.C., Wyngaard J., Izumi Y., Coté O. (1972). Spectral characteristics of surface-layer turbulence. Q. J. R. Meteorol. Soc..

[41] (2007). Eurocode 1: Actions on structures.

[42] Lungu D., Van Gelder P., Trandafir R. (1996). Comparative Study of Eurocode 1, ISO and ASCE Procedures for Calculating Wind Loads. IABSE Report. https://www.researchgate.net/profile/Phajm-Gelder/publication/2467142_Comparative_study_of_Eurocode_1_ISO_and_ASCE_procedures_for_calculating_wind_loads/links/004635325994c4c390000000/Comparative-study-of-Eurocode-1-ISO-and-ASCE-procedures-for-calculating-wind-loads.pdf.

